# p53-Mediated PI3K/AKT/mTOR Pathway Played a Role in Ptox^Dpt^-Induced EMT Inhibition in Liver Cancer Cell Lines

**DOI:** 10.1155/2019/2531493

**Published:** 2019-05-05

**Authors:** Yongli Li, Tingting Wang, Yanjie Sun, Tengfei Huang, Cuiping Li, Yun Fu, Yichun Li, Changzheng Li

**Affiliations:** ^1^Department of Histology and Embryology, Sanquan College of Xinxiang Medical University, Xinxiang, Henan 453003, China; ^2^Experimental Teaching Center of Biology and Basic Medicine, Sanquan College of Xinxiang Medical University, Xinxiang, Henan 453003, China; ^3^Department of Molecular Biology and Biochemistry, Xinxiang Medical University, Xinxiang, Henan 453003, China; ^4^The First Affiliated Hospital of Xinxiang Medical University, Weihui, Henan 453100, China

## Abstract

Epithelial-mesenchymal transition (EMT) involves metastasis and drug resistance; thus, a new EMT reversing agent is required. It has shown that wild-type p53 can reverse EMT back to epithelial characteristics, and iron chelator acting as a p53 inducer has been demonstrated. Moreover, recent study revealed that etoposide could also inhibit EMT. Therefore, combination of etoposide with iron chelator might achieve better inhibition of EMT. To this end, we prepared di-2-pyridineketone hydrazone dithiocarbamate S-propionate podophyllotoxin ester (Ptox^Dpt^) that combined the podophyllotoxin (Ptox) structural unit (etoposide) with the dithiocarbamate unit (iron chelator) through the hybridization strategy. The resulting Ptox^Dpt^ inherited characteristics from parent structural units, acting as both the p53 inducer and topoisomerase II inhibitor. In addition, the Ptox^Dpt^ exhibited significant inhibition in migration and invasion, which correlated with downregulation of matrix metalloproteinase (MMP). More importantly, Ptox^Dpt^ could inhibit EMT in the absence or presence of TGF-*β*1, concomitant to the ROS production, and the additional evidence revealed that Ptox^Dpt^ downregulated AKT/mTOR through upregulation of p53, indicating that Ptox^Dpt^ induced EMT inhibition through the p53/PI3K/AKT/mTOR pathway.

## 1. Introduction

Metastasis is a hallmark of cancer and one of the urgent tasks to be solved in a clinical practice. During metastasis, the malignant cells spread from the primary tumor to distant sites, which cause failure of vital organs, consequently leading to the death of patients. In addition, concomitant to the metastasis, the cells acquire an ability to resist conventional treatments [1]. Therefore, insight into the molecular, cellular, and clinical mechanisms underlying metastatic progression is required in order to develop new diagnostic and therapeutic strategies to prevent and treat metastases. It has been shown that epithelial-mesenchymal transition (EMT) and its reverse process, mesenchymal-epithelial transition (MET), involve a metastatic process, and EMT is a key metastasis-promoting step in many cancers [2], but MET may favor to metastatic inhibition or attenuating drug resistance [3–5]. During EMT, epithelial cells lose their junctions and apical-basal polarity and undergo a change in the signaling programmers, developing an invasive phenotype [6]. The investigations reveal that EMT progression is characterized by loss of the epithelial marker E-cadherin; accordingly, the mesenchymal markers, such as N-cadherin and vimentin, are increased. When tumor cells acquire invasive mesenchymal phenotypes, the motility and invasiveness of them are increased, favoring dissemination from the local site and infiltration into the vascular tumor [7]. Since EMT plays an essential role in cancer metastasis, restoration of MET may efficiently slow dissemination of tumor cells [8]. Therefore, targeting EMT is one of the options in cancer therapy [9–11].

p53 is well characterized as a tumor suppressor gene [12]; the wild-type p53 predominantly functions as a transcription factor, playing important roles in cell growth. However, in most of the tumors, p53 is the most frequently mutated gene, resulting in either loss of function or gain of new function [13, 14]. To restore the function of the p53 pathway, small-molecule inhibitors of MDM2 have been used in cancer treatment strategy. In addition, p53 also plays critical roles in EMT and metastasis [15, 16]; overexpression of p53 in p53-proficient human mammary epithelial cells that have undergone EMT results in their reversion back to an epithelial phenotype. p53 regulating EMT is considered through modulating miRNAs in the mechanism [17, 18]. Furthermore, iron chelator was shown to exert its action of regulation both on p53 and EMT, hinting cellular iron status influenced EMT progress [19]; the details remain elusive.

Since EMT is associated with metastasis and drug resistance [20], EMT inhibition may efficiently slow dissemination of tumor cells [8, 21]. It has been shown that some compounds are capable of inhibiting EMT [22, 23]. Recently, researchers from different laboratories reported that etoposide and other topoisomerase II (Topo II) inhibitors widely used in the clinical practice could reverse EMT [24, 25], revealing another side of those DNA poisoners except their cytotoxicity. In addition, wild-type p53 can also reverse EMT, while the level of p53 was partly dependent on iron status in a cell [26], hinting that an alternative route could be used to alter EMT status through regulating iron homeostasis. Studies have shown that some iron chelators displayed antiproliferative action in the investigated cell lines involved upregulation of p53 [27, 28]. Based on the facts, to achieve an enhanced efficiency of EMT inhibition, synchronously targeting Topo II and p53 might be one of the alternative options in favor of dissemination inhibition of the tumor cell. To achieve the goal, in the present study, a dual functional agent was prepared by combination of the podophyllotoxin (Ptox) structural unit with the dithiocarbamate unit based on a hybridization strategy. The novel compound, di-2-pyridineketone hydrazone dithiocarbamate S-propionate podophyllotoxin ester (Ptox^Dpt^), exhibited significant Topo II inhibition and acted as a p53 inducer. A growth inhibition assay against hepatocellular carcinoma cells *in vitro* revealed that Ptox^Dpt^ displayed a better antiproliferative effect than the parent compounds, 4′-demethylepipodophyllotoxin and etoposide. Moreover, Ptox^Dpt^ exhibited a significant antimetastatic effect, which likely correlated with matrix metalloproteinase (MMP) inhibition and concomitant to the mTOR downregulation. As expected, Ptox^Dpt^ could also reverse TGF-*β*1-induced EMT in hepatocellular carcinoma cells. In addition, ROS are involved in the EMT process [29], but the role of ROS in EMT reversal is not fully determined. We hypothesized that ROS might also play a role in EMT reversion. To address this issue, we measured cellular ROS after stimulation with either TGF-*β*1 or combination with Ptox^Dpt^, revealing that both EMT and EMT reversion involved ROS production. Further study demonstrated that Ptox^Dpt^-induced EMT reversal was through p53-mediated PI3K/AKT/mTOR pathways; this feature is first reported for an etoposide derivative.

## 2. Results

### 2.1. Preparation of Di-2-pyridineketone Hydrazone Dithiocarbamate S-Propionate Podophyllotoxin Ester (Ptox^Dpt^)

Ptox^Dpt^ was prepared through a four-step reaction (Fig.  S1); the detail is described in Materials and Methods. The first three-step reaction for the preparation of di-2-pyridineketone hydrazone dithiocarbamate S-propionate was based on a protocol described previously [30]. The final product, Ptox^Dpt^, was synthesized by mixing 4′-demethylepipodophyllotoxin with di-2-pyridineketone hydrazone dithiocarbamate S-propionate under catalysis of DMAP/DCC in dichloromethane at room temperature. Upon completion of the reaction, the crude product was subjected to flash chromatography. Following the NMR and HRMS characterization, the Ptox^Dpt^ was identified as expected (Fig.  S1).

### 2.2. Topo II Inhibition of Ptox^Dpt^ and Simulation of Binding

**Figure 1:**
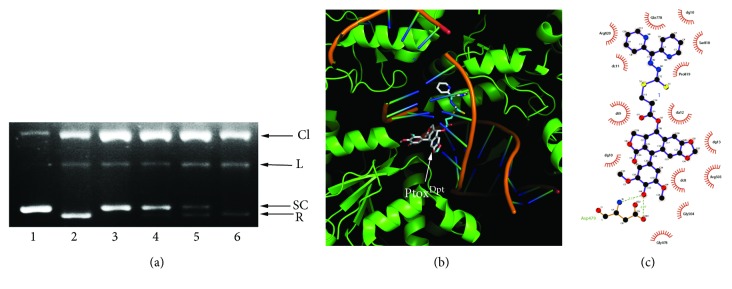
The Topo II inhibition and interaction of Ptox^Dpt^. (a) Topo II inhibition of Ptox^Dpt^. 1: pUC18; 2: nucleic extract plus pUC18; 3: pUC18 and nucleic extract plus 75 *μ*M Ptox^Dpt^; 4: pUC18 and nucleic extract plus 50 *μ*M Ptox^Dpt^; 5: pUC18 and nucleic extract plus 500 *μ*M etoposide; 6: pUC18 and nucleic extract plus DMSO. CL = cleaved; L = linear; S = supercoiled; R = relaxed DNA. (b) Ptox^Dpt^ docked into the Topo II DNA complex. (c) The interactions between Ptox^Dpt^ and nearby residues. The dark green dash lines indicated hydrogen bonds. The eye-like curved dark red lines represented the hydrophobic residues in contact.

Etoposide is a well-known Topo II inhibitor. To understand whether the conjugate, Ptox^Dpt^, retained the same activity as etoposide, we first assessed its inhibitory effect on DNA relaxation. Following a protocol reported previously [31], pUC18 plasmid DNA was incubated with a nuclear extract in the absence or presence of varied concentrations of Ptox^Dpt^, and the reaction products were subjected to agarose gel electrophoresis. As shown in Figure 1(a), Ptox^Dpt^ displayed a certain degree of Topo II inhibition based on EtB prestained agarose gel electrophoresis (Figure 1(a)) as the amount of relaxed DNA decreased compared to that of the control. In addition, Ptox^Dpt^ appeared to be a more potent Topo II inhibitor than etoposide since higher concentration of etoposide was required to achieve the same degree of inhibition. We next determined whether the binding of Ptox^Dpt^ to Topo II was similar to that of etoposide. To do this, a theoretical simulation was performed by using a molecular docking approach. The crystal structure of Topo II of a human (PDB ID: 3QX3) was obtained from the RCSB Protein Data Bank. To ensure the accuracy of our docking protocol, etoposide was redocked into the Topo-DNA complex based on the recommended procedure. The conformation of etoposide in the Topo-DNA complex derived from molecular docking could be almost fully superimposed on the native cocrystallized structure, indicating that the protocol was appropriate. As shown in Fig.  S2A, the conformation of etoposide in the Topo-DNA complex generated from molecular docking was almost fully superimposed on the native cocrystallized one, indicating that the protocol was practical (Fig.  S2A). Thus, the synthesized Ptox^Dpt^ was docked into the Topo II complex following similar protocol (Figure 1(b)), and accordingly, the simulating affinity energy (-13.6 kcal/mol) was obtained. Comparing the simulating affinity energy to that of docked etoposide (-14.8 kcal/mol), a slightly weaker interaction was thus observed. The superimposition of Ptox^Dpt^ on the cocrystallized etoposide is presented in Fig.  S2B, revealing that the replacement of sugar at 4-position with the dithiocarbamate derivative did not lead to significant change in affinity and nearby environment. The interaction of Ptox^Dpt^ with its nearby residues is shown in Figure 1(c), clearly showing that the nature of the interactions was mainly hydrophobic interaction and hydrogen bonds.

### 2.3. Ptox^Dpt^ Inhibits Hepatocellular Carcinoma Cell Proliferation

**Figure 2:**
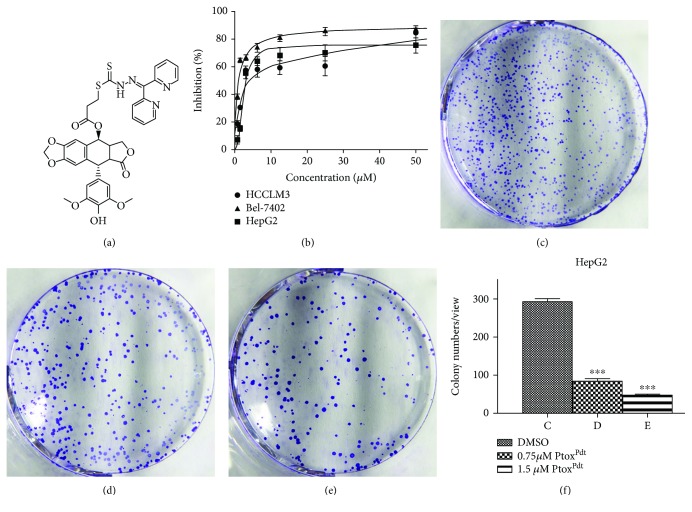
Ptox^Dpt^ induced inhibition in proliferation and colony formation against hepatoma carcinoma cell lines: (a) structure of Ptox^Dpt^; (b) growth inhibition of Ptox^Dpt^ against investigated cell lines with IC_50_ values 4.2 ± 0.2*μ*M for HCCLM3, 1.0 ± 0.1*μ*M for Bel-7402, and 3.0 ± 0.14*μ*M for HepG2 cell, respectively. (c–e) The effect of Ptox^Dpt^ on colony formation of HepG2 cells: (c) 0.75% DMSO; (d) 0.075 *μ*M Ptox^Dpt^; (e) 0.15 *μ*M Ptox^Dpt^. (f) Quantitative analyses were from five visual fields randomly chosen from each well; ^∗∗∗^ *p* < 0.01.

To determine whether cytotoxicity was affected by the structural modification, the effect of Ptox^Dpt^ on the proliferation of three hepatocellular carcinoma cell lines was determined. The dose-response curves for all three cell lines are shown in Figure 2(b). Ptox^Dpt^ displayed significant growth inhibition in all three examined cell lines and was concentration dependent (*p* = 0.012 < 0.05 for HepG2, *p* = 0.008 < 0.01 for Bel-7402, and *p* = 0.0138 < 0.05 for HCCLM3, respectively). However, a slight differential effect on the cell lines was observed; similar growth inhibition was achieved at a lower concentration (IC_50_ ≤ 3*μ*M) for Bel-7402 and HepG2 cells (Figure 2(b)), whereas a higher concentration was required for HCCLM3 cells (Figure 2(b)), indicating that Bel-7402 and HepG2 cells were more sensitive to Ptox^Dpt^. Interestingly, Ptox^Dpt^ exhibited better activity than etoposide in growth inhibition (Fig.  S3). Next, the effect of Ptox^Dpt^ on colony formation of HepG2 cells was further examined. As shown in Figure 2, Ptox^Dpt^ significantly reduced the number of clones formed by HepG2 cells (Figures 2(c)–2(e)); about 65% inhibition at 0.75 *μ*M (*p* < 0.01) and ~84% inhibition at 1.50 *μ*M Ptox^Dpt^ treatment were observed based on quantitative analysis (Figure 2(f)). Taken together, these results demonstrated that Ptox^Dpt^ inhibited the growth of hepatocellular carcinoma cells *in vitro*.

### 2.4. Ptox^Dpt^ Inhibits Cell Migration and Invasion

**Figure 3:**
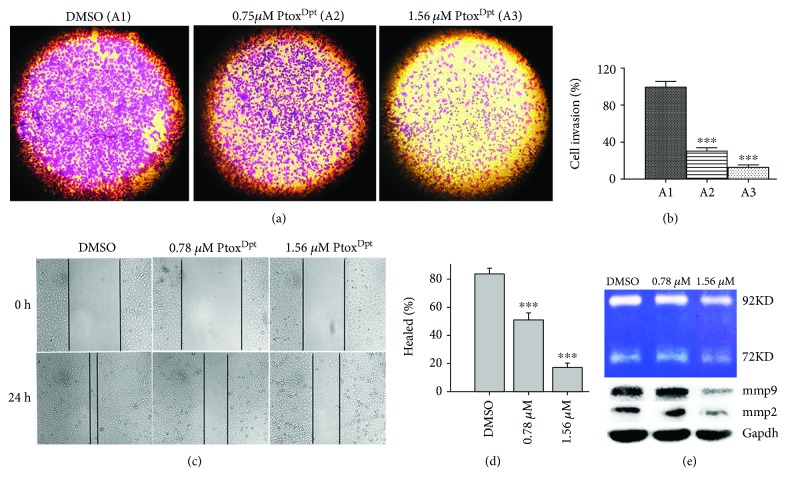
The immigration and invasion inhibition of Ptox^Dpt^ against hepatocellular carcinoma cells. (a) The migratory inhibition of Ptox^Dpt^ against HCCLM3 cells. (b) Quantitative analysis from (a): the invasive cells were stained with crystal violet. The results were expressed as invasive cell numbers per field of view (mean ± 5 SD, *n* = 6). (c) The wounded HCCLM3 cells were treated with 0.0, 0.78, and 1.56 *μ*M Ptox^Dpt^ for 24 h. (d) Quantitative analysis of the width of gaps. (e) Gelatin zymography (A) and Western blotting (B) analyses of matrix metalloprotease inhibition; the condition was as indicated in the experimental section. ^∗∗∗^ *p* < 0.001 compared with the DMSO-treated group.

Both cell invasion and migration are of fundamental importance in tumor metastasis and angiogenesis [32]. The HCCLM3 cell line is widely used in the invasion assay due to a higher potent metastasis than the HepG2 cell; thus, a transwell assay was performed to determine the effect of Ptox^Dpt^ on invasion of HCCLM3 cells. As shown in Figure 3(a), HCCLM3 cells displayed a high invasion capability. In contrast, Ptox^Dpt^ significantly attenuated invasion capacity of the cells in a dose-dependent manner (*p* < 0.05); a quantitative analysis is presented in Figure 3(b). In addition, a wound-healing model is widely used to estimate the migration potential of endothelial cells. Next, the effect of Ptox^Dpt^ on the migration of HCCLM3 cells was determined. As shown in Figures 3(c) and 3(d), the migration of HCCLM3 across the wound space was inhibited by Ptox^Dpt^ in a dose-dependent manner. Furthermore, matrix metalloproteinases (MMPs) as key players are involved in tumor invasion and metastasis [33]; the Ptox^Dpt^-induced migration and invasion inhibition might correlate with MMP inhibition; thus, the Western blotting and gelatin zymography analyses were further conducted. As shown in Figure 3(e), Ptox^Dpt^ treatment significantly reduced both MMP-2 and MMP-9 expression (Figure 3(e), B) and activity (Figure 3(e), A), consistent with a previous report [34].

### 2.5. Ptox^Dpt^ Regulated EMT-Related Proteins

**Figure 4:**
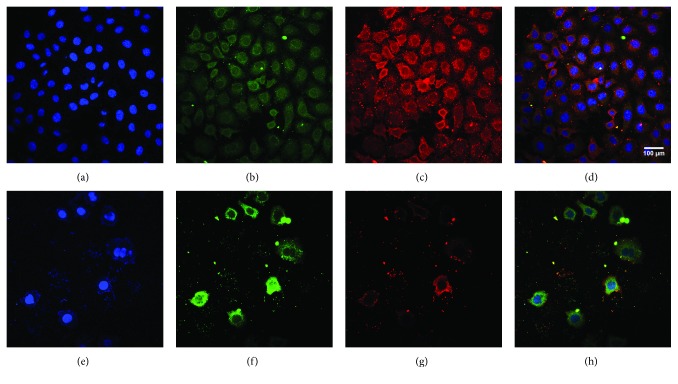
Ptox^Dpt^ inhibited EMT. (a–h) Immunofluorescence analysis of epithelial and mesenchymal markers. (a–d) Control: (a) nuclei in blue; (b) E-cadherin in green; (c) vimentin in red; (d) merge of nuclei, E-cadherin, and vimentin. (e–h) Ptox^Dpt^-treated group: (e) nuclei in blue; (f) E-cadherin in green; (g) vimentin in red; (h) merge of nuclei, E-cadherin, and vimentin. Subject size: 40 × 10 (fluorescence), scale bar: 100 *μ*m.

**Figure 5:**
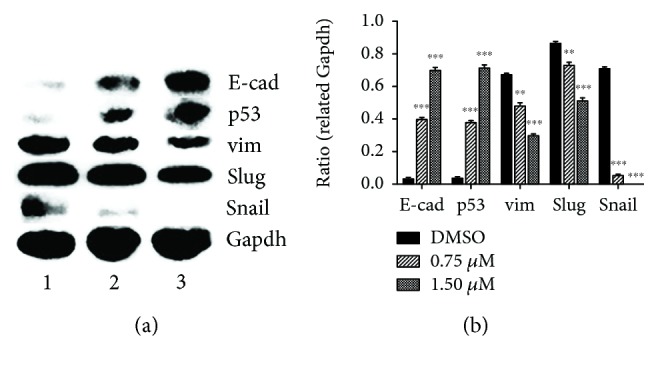
Ptox^Dpt^ regulated EMT-related protein expression. (a) Western blotting analysis of epithelium and mesenchymal markers; (b) quantification analysis of EMT-related proteins was conducted through ImageJ; ^∗∗∗^ *p* < 0.001, ^∗∗^ *p* < 0.005.

Since Ptox^Dpt^ could inhibit invasion and immigration of HCCLM3 cells, it might affect EMT. Considering that the HepG2 cell was more sensitive than HCCLM3, in the following experiments, the HepG2 cell line was chosen. To determine the potential effect of Ptox^Dpt^ on EMT, the alterations in markers of the epithelium (E-cadherin) and mesenchymal cells (vimentin) were investigated. The immunofluorescence technique is widely used to visualize the alteration in membrane proteins; thus, the vimentin (in red) and E-cadherin (in green) were labeled individually (Figure 4); the merged photos could be used to distinguish the difference between the control group and the drug-treated group. Interestingly, Ptox^Dpt^ could decrease the intensity of red fluorescence, i.e., expression of the mesenchymal marker, vimentin (Figures 4(c) and 4(g)); contrarily, it significantly increased the intensity of green fluorescence (or upregulation of expression of the epithelium marker, E-cadherin) compared to the control (Figures 4(b) and 4(f)), indicating that Ptox^Dpt^ could affect EMT transformation of the HepG2 cell (Figures 4(d) and 4(h)). To further support the above conclusion, additional Western blotting analysis was conducted. As shown in Figure 5, a downregulation of vimentin, snail, and slug and upregulation of E-cadherin were observed upon Ptox^Dpt^ treatment, and the significant alterations in epithelium-mesenchymal markers are clearly shown based on the quantitative analysis (*p* < 0.005 or 0.001, Figure 5(b)), corroborating that Ptox^Dpt^ owned the capacity in EMT inhibition. In addition, in view of the critical role of p53 in EMT [17], the EMT inhibition induced by Ptox^Dpt^ might involve p53; thus, the level of p53 was further determined. As expected, Ptox^Dpt^ indeed triggered upregulation of p53 (Figure 5(a)), indicating that p53 may also have a role in the EMT inhibition, consistent with previous reports [17, 18].

### 2.6. The EMT Inhibition Induced by Ptox^Dpt^ Involved ROS Production

**Figure 6:**
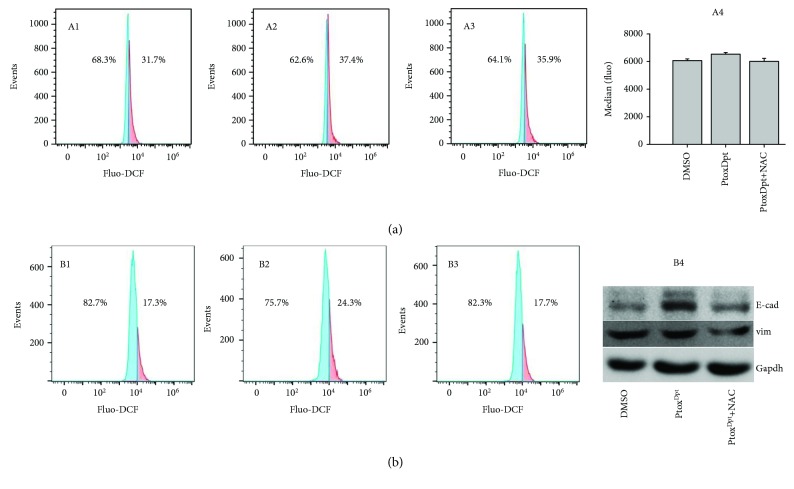
Ptox^Dpt^ treatment induced ROS production at different conditions and alteration in EMT markers. (a) 20 h treatment: A1: 0.75% DMSO; A2: 1.56 *μ*M Ptox^Dpt^; A3: 1.56 *μ*M Ptox^Dpt^ + 1.5 mM NAC; A4: medians of fluorescence in different groups. (b) 2 h treatment in the presence of 10 ng TGF-*β*1: B1: 0.75% DMSO; B2: 1.56 *μ*M Ptox^Dpt^; B3: 1.56 *μ*M Ptox^Dpt^ + 1.5 mM NAC; B4: Western blotting analysis of EMT markers.

The ROS production in EMT transformation has been well documented; however, whether EMT inhibition also involves ROS production remains to be investigated. Since Ptox^Dpt^ could suppress EMT, and lead to p53 upregulation, it might be associated with ROS production for p53 is a redox-active transcription factor, which responds to variety of stresses [35]. To confirm that the upregulated p53 was due to massive ROS production, the abundance of cellular ROS under different conditions was determined. As shown in Figure 6, the treatment of Ptox^Dpt^ caused ~6% increase in higher DCF fluorescence population compared to the control (Figure 6(a), A2) with a short time period of insulting (20 h), but the alteration could be attenuated by the addition of NAC, a ROS scavenger (Figure 6(a), A3). Similar trends were observed from analysis of the fluorescence median (Figure 6(a), A4). Those results might indicate that ROS were indeed involved in Ptox^Dpt^-induced EMT reversal. To corroborate the ROS role in EMT reversal, the HepG2 cells were pretreated with TGF-*β*1 to induce an EMT model and further treated by Ptox^Dpt^ for 2 h; the cells that were treated either by Ptox^Dpt^ or by combination with NAC were subjected to flow cytometry analysis. As shown in Figure 6(b), a 7% increase in higher fluorescence population was observed when the cells were treated by 1.56 *μ*M Ptox^Dpt^, but the addition of NAC eliminated the increase and moved back to the normal state (Figure 6(b), B1–B3), indicating that Ptox^Dpt^-induced EMT inhibition involved ROS production. The additional evidence from Western blotting analysis further supported the above conclusion because the addition of NAC could attenuate the increase of E-cadherin (Figure 6(b), B4). Both conditions hinted that Ptox^Dpt^ could induce ROS production, no matter if TGF-*β*1 existed or not. Furthermore, the ROS were almost not altered after 24 h insulting of Ptox^Dpt^ (Fig.  S4), indicating that a fluctuating ROS model occurred during the EMT transformation.

### 2.7. Ptox^Dpt^-Induced EMT Reversal Was p53 Dependent

**Figure 7:**
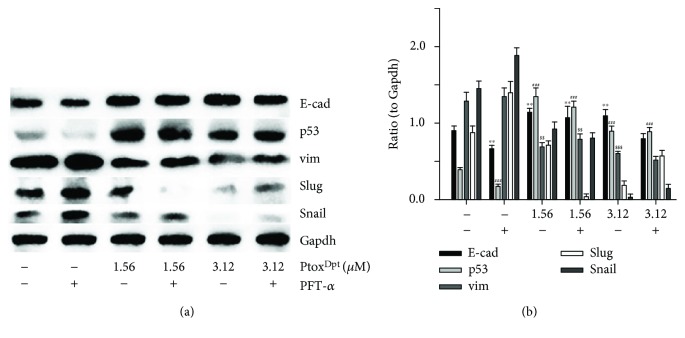
Ptox^Dpt^-induced EMT reversion was p53 dependent. (a) Western blotting analyses of p53 and epithelium-mesenchymal proteins. (b) Quantitative analysis of proteins during EMT reversal induced by Ptox^Dpt^. ^∗∗^ *p* < 0.05 for E-cad; ^##^ *p* < 0.05, ^###^ *p* < 0.01 for p53; ^$$^ *p* < 0.05, ^$$$^ *p* < 0.01 for vimentin.

Ptox^Dpt^ led to upregulation of p53 (Figure 5(a)), hinting that p53 might be involved in the EMT reversal. To corroborate the role of p53 in EMT inhibition induced by Ptox^Dpt^, a p53 inhibitor, PFT-*α*, was used to downregulate p53; then, the expressions of E-cadherin, vimentin, slug, and snail were determined. As shown in Figure 7, PFT-*α* indeed upregulated vim, slug, and snail and downregulated E-cadherin, and combination treatment of Ptox^Dpt^ with PFT-*α* significantly attenuated the expression of mesenchymal proteins and enhanced epithelium protein, E-cadherin (Figure 7(a)), indicating that p53 played a crucial role in EMT transition.  Figure 7(b) showed quantitative analyses for the relative proteins under different treatments.

### 2.8. Ptox^Dpt^ Attenuated TGF-*β*1-Induced EMT

**Figure 8:**
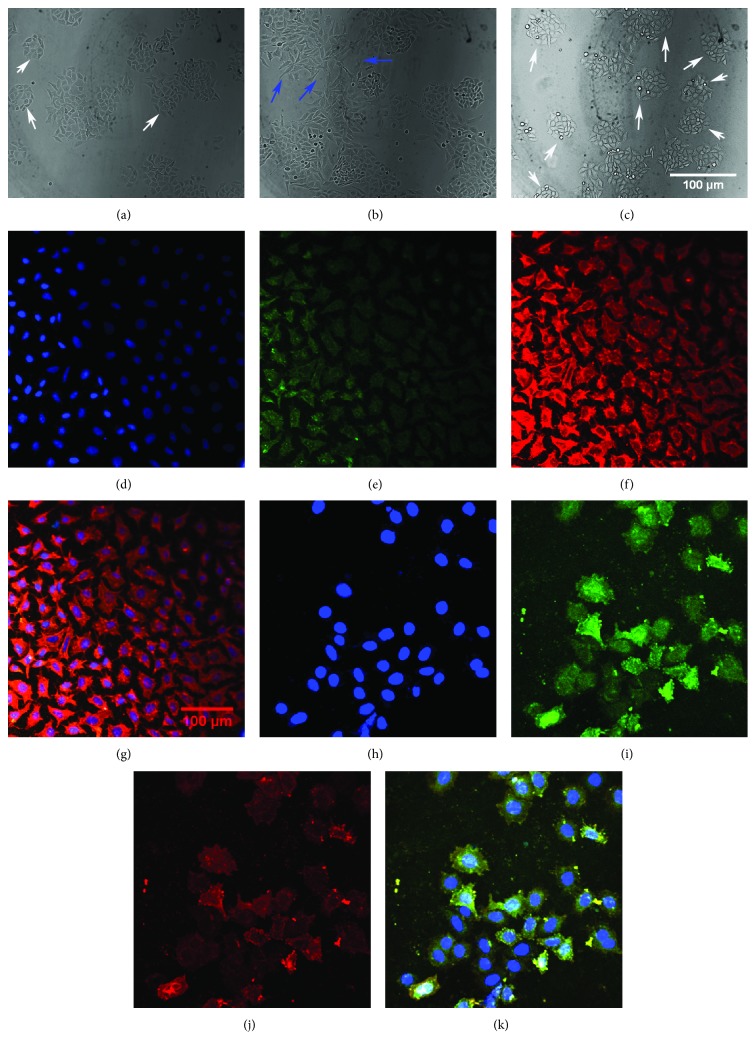
Ptox^Dpt^ inhibited EMT. (a–k) Immunofluorescence analysis of epithelial and mesenchymal markers. (a–d) Control: (a) nuclei in blue; (b) E-cadherin in green; (c) vimentin in red; (d) merge of nuclei, E-cadherin, and vimentin. (e–h) Ptox^Dpt^-treated group: (e) nuclei in blue; (f) E-cadherin in green; (g) vimentin in red; (h) merge of nuclei, E-cadherin, and vimentin. Blue arrow: undergoing EMT; white arrow: original or back to the original state. Subject size: 40 × 10 (fluorescence), scale bar: 100 *μ*m.

**Figure 9:**
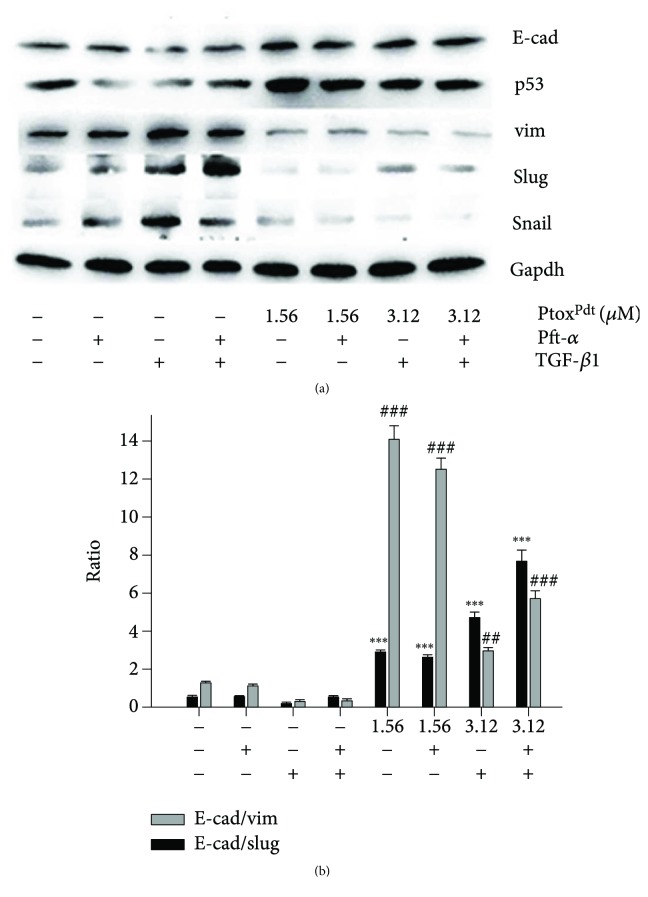
Ptox^Dpt^ counteracted EMT induced by TGF-*β*1 in HepG2 cells. (a) Western blotting analysis; (b) quantitative comparison of the ratio of E-cad/vim (slug) in different conditions. The average ratio was from three independent experiments.

TGF-*β*1 as the most used EMT inducer can induce a mesenchymal phenotype in many cell lines, including the HepG2 cell line [36]. To verify the effectiveness of Ptox^Dpt^ in EMT inhibition, the HepG2 cells were pretreated by TGF-*β*1; as shown in Figure 8(b), spindle-shaped, fibroblast-like HepG2 cells were observed after TGF-*β*1 treatment, indicating that HepG2 cells were undergoing EMT, and the EMT model was successfully established [37]. Next, the cells were subjected to combination treatment of Ptox^Dpt^ and TGF-*β*1; interestingly, Ptox^Dpt^ could restore the cells back to the original state (Figure 8(c)), supporting the role of Ptox^Dpt^ in EMT. To gain more insight into EMT, immunofluorescence analysis was further conducted.  Figure 8 clearly showed that Ptox^Dpt^ could decrease the intensity of red fluorescence of vimentin in the presence of TGF-*β*1 (Figures 8(f) and 8(j)) and accordingly increase the intensity of green fluorescence of E-cadherin (Figures 8(e) and 8(i)) compared to the control, further supporting that Ptox^Dpt^ could inhibit EMT. The additional evidence from Western blotting also supported the above conclusion for the expression of the epithelial marker, E-cadherin, and the mesenchymal markers, vimentin, slug, and snail were all altered when the HepG2 cells were treated by either TGF-*β*1 (Ptox^Dpt^) or combination of TGF-*β*1 and Ptox^Dpt^ (Figure 9). There was a significant increase in the ratio of E-cad/vim (3 folds) comparing Ptox^Dpt^ to TGF-*β*1 treatment, a similar trend in the ratio of E-cad/slug (6 folds, Figure 9(b)); those indicated that an epithelial characteristic was significantly enhanced after Ptox^Dpt^ treatment, demonstrating that Ptox^Dpt^ was indeed able to counteract TGF-*β*1 action in EMT induction. The p53 inhibitor, PFT-*α*, like TGF-*β*1 led to p53 downregulation (Figure 9(a)), which accordingly caused mesenchymal characteristic enhancement, but ratios of E-cad/vim (slug) were not significantly altered except p53 downregulated in the combination treatment of PFT-*α* (or TGF-*β*1) with Ptox^Dpt^, implying that Ptox^Dpt^ owned a powerful ability in EMT inhibition.

### 2.9. Ptox^Dpt^ Induced Invasion Inhibition and EMT Reversal Involved in AKT/mTOR Pathways

**Figure 10:**
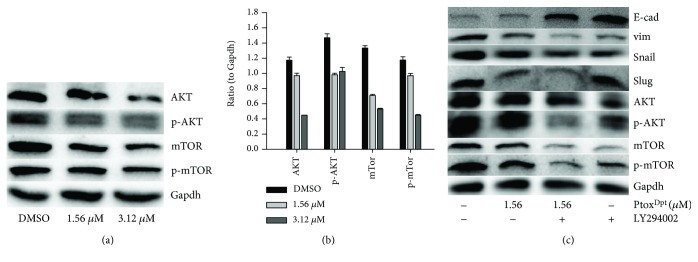
Ptox^Dpt^ regulation on the AKT/mTOR signal pathway: (a) Western blotting analysis of the AKT/mTOR signal pathway. The condition was as indicated; (b) quantitative analysis of the AKT/mTOR signal pathway; (c) the AKT/mTOR signal pathway involved EMT transformation induced by Ptox^Dpt^. ^∗∗∗^ *p* < 0.01; ^∗∗^ *p* < 0.05.

Inhibition of mTORC1 and mTORC2 could attenuate migration and invasion [38]; Ptox^Dpt^-induced migration and invasion inhibition might involve mTOR inhibition or stem from the alteration of the PI3K/AKT/mTOR pathway [34]. Thus, the levels of AKT, phospho-AKT (as a measure of AKT activation), and mTOR were firstly determined by Western blotting. As shown in Figure 10(a), both AKT and phosphorylated AKT (p-AKT) were decreased after Ptox^Dpt^ treatment, hinting that downregulation of p-AKT may stem from the downregulated AKT. A similar trend for mTOR, a downstream target of AKT, was also observed, indicating that the metastasis and invasion inhibition correlated with downregulation of mTOR that led to lower abundances of mTORC1 and mTORC2 complexes. The quantitative analysis of the proteins is shown in Figure 10(b); clearly, Ptox^Dpt^-induced downregulation of both AKT and mTOR had significant statistical significance (*p* < 0.05 or 0.01). On the other hand, in addition to migration and invasion inhibition, Ptox^Dpt^ also inhibited EMT, whether the PI3K/AKT/mTOR pathway was similarly involved in the EMT inhibition. To this end, the level of markers of the epithelium and mesenchymal cells, as well as AKT/mTOR, in the absence or presence of AKT inhibitor, LY294002, was further determined by Western blotting. As shown in Figure 10(c), both Ptox^Dpt^ and LY294002 downregulated vimentin (snail and slug) and contrarily upregulated E-cadherin, indicating that they acted in a similar way in EMT inhibition. Moreover, Ptox^Dpt^ also downregulated AKT and mTOR as LY294002 did (Figure 10(a) and 10(c)), indicating that Ptox^Dpt^-induced EMT inhibition involved the PI3K/AKT/mTOR pathway.

## 3. Discussion

Epithelial-mesenchymal transition (EMT) involves the metastatic process of cancer; targeting EMT is one of the options in cancer therapy; thus, the novel EMT reversal agents are required. It has been demonstrated that the alterations in abundance of epithelial-mesenchymal proteins, such as E-cadherin, N-cadherin, and vimentin determine EMT status. On the other hand, to keep cancer cells thriving, higher abundances of DNA topoisomerases (Topo) and iron are needed; thus, either downregulation (or inhibition) of topoisomerase or depletion of iron can slow down the proliferation of cancer cells. Recent studies demonstrated that some Topo II inhibitors, including etoposide, could inhibit EMT and attenuate metastasis [24, 25], which hinted that etoposide derivatives can be used as a library of the EMT inhibitor. However, etoposide is derived from 4′-demethylepipodophyllotoxin (DMEP); thus, DMEP can be used as a basic structural unit to synthesize potent EMT inhibitors. Although a number of modifications at position 4 in DMEP have been conducted, including esterification and amination [39–42], the effect of DMEP derivatives on EMT transformation was not fully determined. In addition, the EMT inhibition could be achieved by introduction of wild-type p53 [15, 16]. Furthermore, some iron chelators could also modulate both p53 and EMT [27, 28]; this implied that iron chelator can function as partial p53 inducer to counteract EMT. For this reason, in the present study, we constructed a novel EMT reversal agent that hybridized the DMEP unit with iron chelator, dithiocarbamate unit, to achieve efficient EMT inhibition (Fig.  S1). As expected, Ptox^Dpt^ inherits the feature of DMEP and exhibits enhanced activity in Topo II inhibition compared to etoposide (Figure 1). A theoretical simulation revealed that Ptox^Dpt^ like etoposide was located in the catalytic center of the DNA Topo complex (Fig.  S3), supporting that Ptox^Dpt^ was a good Topo II inhibitor except EMT inhibition. In addition, Ptox^Dpt^ exhibited significant growth inhibition (Figure 2) and migration and invasion inhibition partly correlated with downregulation (or inactivation) of MMPs (Figure 3), in accordance with a previous report [43].

It has been shown that epithelial-mesenchymal transition (EMT) is associated with increase of matrix metalloproteases (MMPs) [38]; downregulation of MMP may inhibit EMT. Since Ptox^Dpt^ induced a downregulation of MMPs, it might inhibit EMT. To test the hypothesis, the alterations in epithelial-mesenchymal markers both in immunofluorescence and in the protein level were investigated. Immunofluorescence analyses in Figure 4 revealed that Ptox^Dpt^ inhibited EMT through upregulation of E-cadherin and downregulation of vimentin; additional evidence from Western blotting (Figure 5) supported the above deduction, similar to that reported previously [44]. As expected, the introduction of dithiocarbamate in Ptox^Dpt^ led to an upregulation of p53, which might partly stem from depletion of cellular iron. However, other factors cannot be ruled out for p53 are responsible for different stresses. It was reported that Topo II inhibition mediated oxidative stress-involved ROS production [45]; a similar situation may occur in Ptox^Dpt^-treated cells. In addition, it was well documented that TGF-*β*1-induced EMT involved ROS production [46, 47]; however, whether the EMT inhibition also involved ROS production remained to be determined. To test the hypothesis, Ptox^Dpt^-induced ROS production was assayed either in the absence or in the presence of TGF-*β*1 (Figures 6(a) and 6(b)); the results clearly showed that EMT inhibition indeed involved ROS production, which also correlated with upregulation of E-cadherin (or downregulation of vimentin, Figure 6(b), B4). To further support the above conclusion, the addition of NAC could attenuate EMT inhibition induced by Ptox^Dpt^ (Figure 6(b), B4), indicating that ROS played a role in the EMT inhibition. Furthermore, this also hinted that the upregulated p53 might be due to ROS production.

It should be noted that the excess ROS generation only occurred in the initial stage in the presence of TGF-*β*1, almost no change for ROS after 24 h (Fig.  S4); this situation in ROS production was consistent with that described previously [45, 48]. To respond to the ROS production, p53 was upregulated; contrarily, addition of the p53 inhibitor, PFT-*α*, significantly attenuated the effect of Ptox^Dpt^ on epithelium-mesenchymal markers, indicating that p53 played a role in Ptox^Dpt^-induced EMT inhibition (Figure 7). In addition, the additional evidence also showed that Ptox^Dpt^ could suppress TGF-*β*1-induced EMT both from immunofluorescence and Western blotting (Figures 8 and 9), further supporting that Ptox^Dpt^-induced p53 upregulation played an important role in EMT inhibition.

The PI3K/AKT/mTOR pathway plays a critical role in the proliferation, apoptosis, angiogenesis, and metastasis of tumor development [49, 50]. Ptox^Dpt^ exhibited significant invasion and EMT inhibition; we thus questioned whether Ptox^Dpt^-induced EMT inhibition was through the PI3K/AKT/mTOR pathway. To this end, the level of the PI3K/AKT/mTOR pathway was determined; as expected, Ptox^Dpt^ could downregulate both AKT and mTOR expression (Figures 10(a) and 10(b)), suggesting that the action of Ptox^Dpt^ on the HepG2 cell might involve the PI3K/AKT/mTOR pathway. To corroborate the involvement of the PI3K/AKT/mTOR pathway in Ptox^Dpt^-induced EMT inhibition, an AKT inhibitor, LY294002, was used as the positive control; the data clearly showed that both Ptox^Dpt^ and LY294002 achieved EMT inhibition (Figure 10(c)), indicating that Ptox^Dpt^-induced EMT inhibition involved the PI3K/AKT/mTOR pathway.

In conclusion, the Ptox^Dpt^ exhibited diverse functions in both Topo II and MMP inhibition. In addition, Ptox^Dpt^ also inhibit EMT, which may be achieved through the p53/PI3K/AKT/mTOR axis. Importantly, ROS played a role both in EMT and EMT reversal. Ptox^Dpt^ acted as “fighting fire with fire” in the EMT reversal process. Taken together, our findings indicate that Ptox^Dpt^ is a promising antitumor drug for possible use in chemotherapy. However, extensive investigations, both *in vitro* and *in vivo*, are required in future studies.

## 4. Materials and Methods

### 4.1. Materials

MTT, PFT-*α*, TGF-*β*1, di-2-pyridylketone, RPMI-1640, LY294002, and other chemicals were purchased from Sigma-Aldrich (Shanghai, China). Fetal bovine serum was purchased from Every Green Zhejiang Tianhang Technology Co. Ltd. (Hangzhou, China). Antibodies of vimentin, slug, snail, and p53 were purchased from Boster (Wuhan, China). Antibodies of AKT, p-AKT, mTOR, p-mTOR, E-cadherin, and Gapdh were purchased from EnoGene (Nanjing, China). 4′-Demethylpodophyllotoxin was purchased from Shanghai PureOne Biotechnology (Shanghai, China).

#### 4.1.1. Preparation of 2,2′-Dipyridineketone Hydrazone Dithiocarbamate S-Propionic Acid Podophyllotoxin Ester (Ptox^Dpt^)

The Ptox^Dpt^ was prepared by using a four-step reaction: preparation of 2,2′-dipyridineketone hydrazone dithiocarbamate S-propionic acid (compound III, Fig.  S1) engaged a three-step reaction that was reported previously [30]. The Ptox^Dpt^ (compound IV, Fig.  S1) was prepared by reacting compound III with 4′-demethylpodophyllotoxin with DCC/DMAP catalysis in absolute CH_2_Cl_2_. TLC was traced during the period of reaction. ^1^HNMR (Bruker, DMSO-d_6_, ppm): 14.98(s,1H), 8.87(d, 1H, *J* = 4 Hz), 8.63(m, 1H, *J* = 4 Hz), 8.01(m, 3H, *J* = 4, 8 Hz), 7.54 (m, 3H, *J* = 4,8 Hz), 7.00(s, 1H), 6.95(s, 1H), 6.76(s, 2H), 6.56(s, 2H), 6.33(s,1H), 6.01(s,3H), 5.64(d, 1H, *J* = 8 Hz),4.75(dd, 1H, *J* = 4 Hz), 4.52(dd, 1H, *J* = 4 Hz), 4.36(d,1H,*J* = 8 Hz), 4.16(dd, 1H, *J* = 8 Hz), 3.69(s, 6H), 2.93(d, 2H, *J* = 4 Hz). ^13^CNMR(Bruker, 100 MHz, DMSO-d_6_):199.77, 179.25, 175.24, 169.82, 155.02, 151.93, 149.24, 148.94, 147.66, 146.79, 146.73, 145.23,140.66, 138.35, 137.91, 133.26, 130.81, 128.12, 126.94, 125.85, 124.97, 124.02, 109.49, 106.48, 104.92, 101.32, 68.00, 65.95, 65.39, 60.23, 56.47, 56.26, 45.46, 43.15, 33.20, 28.97. ESI-MS (C_36_H_32_N_4_O_9_S_2_K): m/z: 767.1268 (M+K, Calcd: 767.1248).

#### 4.1.2. DNA Topo II Activity Assay

The assay of inhibition of Ptox^Dpt^ on DNA Topo II activity was conducted based on the protocol described previously [31]. To initiate the enzymatic reaction, nuclear extract (0.4 *μ*g) was added to the Topo reaction buffer (10 mM Tris-HCl (pH 7.5), 1 mM EDTA, 1 mM ATP, 150 mM NaCl, 0.1% BSA, and 5% glycerol) that contained 0.4 *μ*g supercoiled pUC18 plasmid DNA and 1-3 *μ*L of Ptox^Dpt^ (1 mM in 8% DMSO) in a final volume of 20 *μ*L. Following an additional incubation at 37°C for 30 min., 5 *μ*L of stopping buffer (10% SDS, 0.025% bromophenol blue, and 10% glycerol) was added to terminate the reaction. The resulting products were separated by electrophoresis using a 1% agarose gel in a TBE buffer (89 mM Tris-HCl, 89 mM boric acid, and 62 mM EDTA) containing 0.1% SDS and ethidium bromide (0.5 *μ*g/mL) at 45 V for 3 h. The bands were visualized on a Tocan 360 gel scanner (excited at ~340 nm) (Shanghai Tiancheng Technology Inc., China). The assay was performed in duplicate.

#### 4.1.3. Molecular Docking

To simulate the potent interaction between Ptox^Dpt^ and Topo II, the PDB file of the structure of human type II Topo (3QX3) was downloaded from the RCSB Protein Data Bank. The structure of Ptox^Dpt^ was generated by ChemDraw. Then, the 3QX3 and Ptox^Dpt^ in PDB were transformed in PDBQT format by the AutoDock Tool using the default parameters. PyMOL and LigPlot were used to display the conformation and interactions [51, 52].

Molecular docking studies were performed by using AutoDock Vina [53]. To optimize the docking parameters, the cocrystallized etoposide was extracted and redocked into the active sites of 3QX3 with various parameters. The grid box size for Ptox^Dpt^ was set to 22, 24, and 28 for the *x*-, *y*-, and *z*-axes, respectively.

#### 4.1.4. Cytotoxicity Assay (MTT Assay)

The proliferative inhibition of the agent was determined by the MTT method as described previously [30]. Briefly, the equivalent cells of Bel-7402 (or HepG2, HCCLM3, 5 × 10^3^/mL) were seeded into a 96-well plate, and the varied concentrations of Ptox^Dpt^ were added to the wells after the cells were adhered. Following 48 h incubation at 37°C in a humidified atmosphere of 5% CO_2_, 10 *μ*L of MTT solution (5 mg/mL) was added into each well, and a further incubation was conducted. Finally, 100 *μ*L DMSO was added to each well to dissolve the formazan crystals after removing the cell culture. The measurement of the solution absorbance was performed on a microplate reader (MK3, Thermo Scientific) at 570 nm. The percent absorbance inhibition that correlates with percent growth inhibition was obtained. The same assay was performed in triplicate.

#### 4.1.5. Plate Clone Formation Assay

The HepG2 cells in the exponential phase were trypsinized and seeded in 6-well plates at the density of 500 cells/well. Ptox^Dpt^ at dose of 1/40 or 1/20 IC_50_ was added. Fourteen days later, colonies were fixed in 3.7% paraformaldehyde, stained with 0.1% crystal violet. Colonies containing 50 cells at least were counted under an inverse microscope (Nikon, Tokyo, Japan), and the clone numbers were analyzed subsequently.

#### 4.1.6. Migration Assay

The inhibition of tumor cell migration by Ptox^Dpt^ was determined using a wound-healing migration assay [54]. Briefly, HCCLM3 cells were allowed to grow to full confluence in 6-well plates, after which “wounds” were created using a sterile pipette tip. Following this procedure, the cells were rinsed twice with PBS to remove unattached cells. Fresh medium containing 10% fetal bovine serum and various concentrations of Ptox^Dpt^ was then added. The cells were photographed (time 0). After 24 h incubation at 37°C, the cells were photographed again (24 h).

#### 4.1.7. Invasion Assay

As described previously [55], transwell chambers (Corning) with 8 *μ*m pore membranes coated with Matrigel were used to perform the invasion assay. Briefly, after overnight pretreatment with Ptox^Dpt^ in a 6-well plate, the HCCLM3 cells were starved for 12 h in serum-free medium. Following this, the cells were collected and resuspended as a single cell suspension. In total, 3 × 10^4^ cells in 100 *μ*L of serum-free medium were added to the upper chamber, and 600 *μ*L of complete medium was added to the lower chamber. Following incubation for 18 h at 37°C, the invading cells were fixed in 4% paraformaldehyde, stained with 0.1% crystal violet, and photographed under a microscope (or counted manually). The percentage of inhibition was expressed using control wells as 100% (Fig.  S3).

#### 4.1.8. Gelatin Zymography Assay

Gelatin zymography was performed as previously described [56]. Conditioned media were collected from HCCLM3 cells after culture for 24 h in serum-free medium with or without Ptox^Dpt^. After collection, the media were centrifuged to pellet any insoluble material. The protein concentration in the conditioned media was quantified using the Bradford method. Equal amounts of conditioned media were mixed with sample buffer and applied to 10% SDS polyacrylamide gel copolymerized with 1 mg/mL of gelatin. After electrophoresis, the gel was incubated in renaturing buffer (50 mM Tris-HCl, pH 7.5, 2.5% Triton X-100, 200 mM NaCl, 10 mM CaCl_2_, and 1 *μ*M ZnCl_2_), followed by a 24 h incubation with developing buffer (50 mM Tris base, 200 mM NaCl, 10 mM CaCl_2_, 1 *μ*M ZnCl_2_, and 0.02% NaN_3_). The gel was then stained with 0.25% Coomassie blue R-250 for 1 hour and destained with 10% methanol with 5% acetic acid. Clear bands against a dark blue background indicated where the protease had digested the gelatin and were taken to be indicative of protease activity.

#### 4.1.9. Immunofluorescence Analysis

HepG2 cells were first cultured in a 24-well plate with cover glass overnight. Following Ptox^Dpt^ treatment for additional 24 h, cells were first fixed with 4% paraformaldehyde in PBS for 15 min at 37°C and then permeabilized with 0.5 % Triton X-100 in PBS for 20 min. After blocking with 3% BSA in PBS for 60 min, the cells were incubated with combined vimentin with E-cadherin (EnoGene) primary antibody based on protocol recommended by the company; at 4°C, the plate was shaken overnight. Next, removing the primary antibodies and washing with PBS, the cells were further incubated with fluorescence-labeled secondary antibody for 3 h at room temperature. After removing the secondary antibody, the cells were further counterstained with DAPI. Finally, a confocal laser scanning microscope (Nikon Eclipse Ts2, Japan) was used to visualize the cells; the representative cells were selected and photographed.

#### 4.1.10. Flow Cytometry Analysis of Cellular ROS

HepG2 cells were placed in a six-well plate, once the cells were adhered and subjected to treatment with Ptox^Dpt^ or other agent for 24 h. Next, the cells were treated by trypsin digestion and collected. Following washing, the cells were stained with H_2_DCF-DA (Dojindo Laboratories, Japan). The intracellular ROS assay was conducted on a flow cytometry (Becton-Dickinson, USA).

#### 4.1.11. Western Blotting Analysis

The protein extracts were prepared based on the company recommended protocol (Solarbio). Briefly, the HepG2 cells treated with or without Ptox^Dpt^ were scraped in lysis buffer (RIPA lysis buffer), and the cell suspension in the EP tube was incubated on ice for 30 min and mixed by turning upside down occasionally, followed by centrifugation at 14,000 ×g. The clear supernatant was stored at -80°C. Protein concentration was determined using a colorimetric BCA assay. Proteins (20 *μ*g) were loaded on a 13% sodium dodecyl sulfate-polyacrylamide gel at 120 V (20 mA) for 2 h. The separated proteins were subsequently transferred onto a PVDF membrane at 120 V (200 mA) for 90 min. The membrane was washed with Tris-buffered saline (TBS) and then blocked for 2 h in TBS containing 0.1% Tween-20 and 5% nonfat skimmed milk. The membrane was incubated at 4°C overnight with the appropriate primary antibody used at a dilution recommended by the company. The membrane was then washed several times with TBST and subsequently incubated with the appropriate HRP-conjugated secondary antibody for 2 h at room temperature. Finally, the protein bands were detected using a super sensitive ECL solution (Boster Biological Technology Co. Ltd.) and visualized on a Syngene G:BOX imager (Cambridge, United Kingdom). Quantification of protein band intensities was performed using ImageJ software.

#### 4.1.12. Statistical Analysis

Results are presented as the mean ± SEM. Comparisons between two groups were carried out using two-tailed Student's *t*-test. Comparisons between multiple groups were performed by one-way ANOVA with Dunnett post hoc correction. All statistical tests were conducted by using IBM SPSS Statistics (version 19 software). *p* < 0.05 was accepted as significant.

## Data Availability

The data used to support the findings of this study are included within the article.

## Supplementary Materials

Supplementary Materials Fig. S1: synthetic route of Ptox^Dpt^, the condition as indicated in the scheme. The data of NMR and HRMS are shown in Materials and Methods. Fig. S2: molecular simulation of etoposide and Ptox^Dpt^. (A) Comparison of docked etoposide with crystalized etoposide in the DNA topoisomerase complex; (B) comparison of docked etoposide with docked Ptox^Dpt^ in the DNA topoisomerase complex. Fig. S3: proliferation inhibition of etoposide against HepG2 (A) and Ptox^Dpt^ against the normal human hepatic cell line LO2 cell (B). Fig. S4: ROS production induced by Ptox^Dpt^ after 24 h during EMT reversal: (A) DMSO, (B) 156 *μ*M Ptox^Dpt^, and (C) 156 *μ*M *Ptox* ^*Dpt*^ + 3 mM NAC.
