# Supplementary material for: p53-Mediated PI3K/AKT/mTOR Pathway Played a Role in PtoxDpt-Induced EMT Inhibition in Liver Cancer Cell Lines
Source: Oxid Med Cell Longev. 2019 May 5;2019:2531493. doi: 10.1155/2019/2531493 (PMC6525883; doi:10.1155/2019/2531493)
Supplement: Supplementary Materials — Fig. S1: synthetic route of PtoxDpt, the condition as indicated in the scheme. The data of NMR and HRMS are shown in Materials and Methods. Fig. S2: molecular simulation of etoposide and PtoxDpt. (A) Comparison of docked etoposide with crystalized etoposide in the DNA topoisomerase complex; (B) comparison of docked etoposide with docked PtoxDpt in the DNA topoisomerase complex. Fig. S3: proliferation inhibition of etoposide against HepG2 (A) and PtoxDpt against the normal human hepatic cell line LO2 cell (B). Fig. S4: ROS production induced by PtoxDpt after 24 h during EMT reversal: (A) DMSO, (B) 156 μM PtoxDpt, and (C) 156 μM Ptox Dpt + 3 mM NAC. [file 2531493.f1.pdf]

Supplementary Materials

Synthetic route of di-2,2'-pyridineketone hydrazone dithiocarbamate S-propionic acid podophyllotoxin ester (Ptox^{Dpt})

The details for Ptox^{Dpt} preparation as described in section of Materials and methods

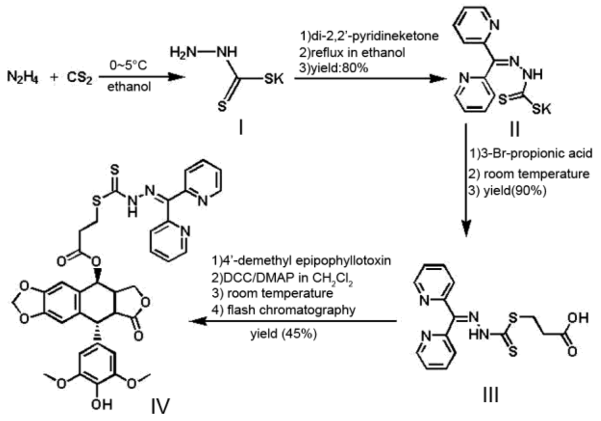

Fig. S1: synthetic route of Ptox^{Dpt}, the condition as indicated in the scheme. The data of NMR and HRMS are shown in Methods and Materials section

Conformation comparison of etoposide with Ptox^{Pdt} in catalytic center of DNA Topoisomerase II

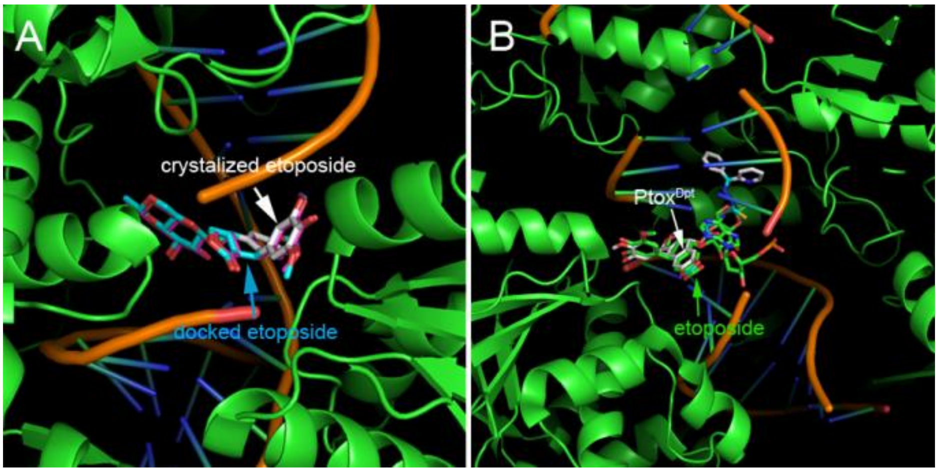

Fig. S2: Molecular simulation of etoposide and Ptox^{Pdt}. (A) Comparison of docked etoposide with crystalized etoposide in DNA-topoisomerase complex; (B) Comparison of docked etoposide with docked Ptox^{Pdt} in DNA-topoisomerase complex.

Comparison of etoposide with Ptox^{Pdt} in growth inhibition against indicated cell lines

The protocol used was similar to that in section of Materials and methods, 4.4 Cytotoxicity assay (MTT assay).

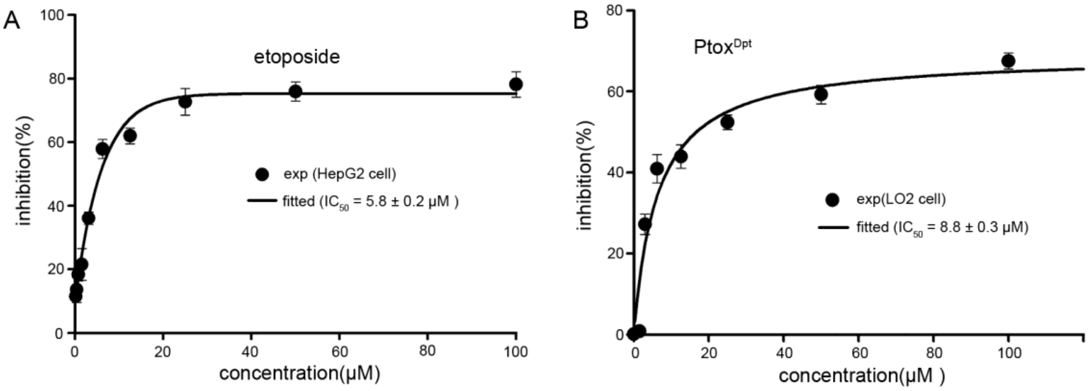

Fig. S3: Proliferation inhibition of etoposide against HepG2(A), and Ptox^{Dpt} against normal human hepatic cell line LO2 cell (B).

Ptox^{Pdt} induced ROS during the time period

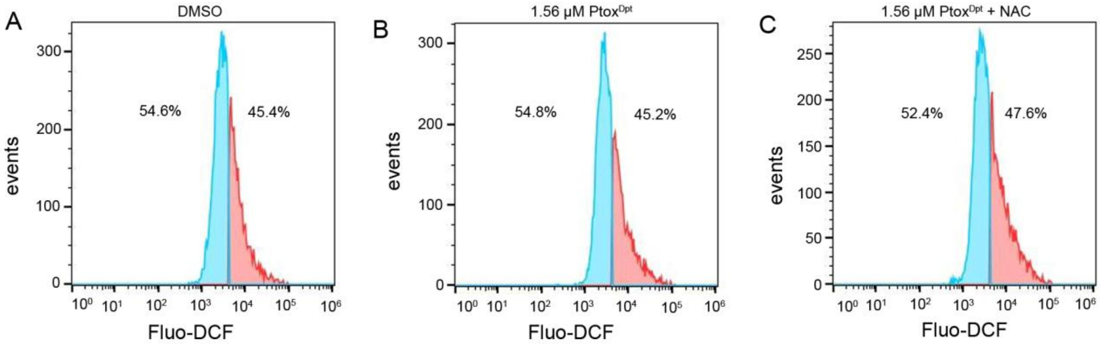

Fig. S4: ROS production induced by Ptox^{Dpt} after 24 h during EMT reversal: (A) DMSO; (B) 1.56 μM Ptox^{Dpt}; (C) 1.56 μM Ptox^{Dpt} + 3 mM NAC.
